# Obituary of Dr. Hales

**Published:** 1835

**Authors:** 


### X. OBITUARY.

Died, at Fort Gibson, Arkansas Territory, on the 30th of January last, Dr. Samuel Watson Hales, Assistant Surgeon, in the United States Army.

As this young gentleman was our private pupil, nearly up to the time of entering the Army, we cannot forego the melancholy pleasure, of recording our testimony to his merits.

The deceased was a son of Mr. Charles Hales, and born in this city, on the 22d July, 1811. Receiving a respectable, though not liberal education in the Cincinnati College, he commenced the study of Medicine in the year 1829.

In the winter of 1831–2, he attended a course of lectures in the Medical College of Ohio; and in the following summer, resolving to commence the practice of his profession, he settled in Louisville. A previous desire to enter the army, however, made him look upon that settlement as temporary; and, accordingly, it was not long before he succeeded in obtaining an order to repair to Jefferson Barracks, for a probationary examination before an army Medical Board. The result was his appointment by the Secretary at War.

During his pupilage, the conduct of Mr. Hales was characterized, by diligence, ambition, enterprize, general propriety and great pride of character; qualities which seemed peculiarly to fit him for the military or naval service.

Immediately after his appointment, he was ordered on the ill-fated expedition, against the Indians high up the Arkansas river; and placed in situations, which at once devolved upon him the duties and responsibilities of a regimental surgeon. The medical history of that unfortunate and disastrous campaign, has not yet been written, but whenever it may be, we shall see, that the sufferings of those who conducted it, and the destruction of life from disease, have never been equalled, in the military operations of our country. As a specimen, we shall transcribe a part of one of the letters of Dr. Hales to his father, dated at Camp Washita, on the S. W. frontier of the United States, July 29th, 1834.

"I started from Camp Canadian, from which my last letter was dated, on the 9th inst. the following Sunday, and arrived here on Wednesday about noon, having met a second express coming for me. Upon my arrival I found this camp in a most miserable situation; out of about 98 men 85 were sick, there was no surgeon, and but little medicine. The assistant surgeon who had been here, had departed a few days before, because it was reported that I was to be here.

The second night after my arrival, I was ordered over to Colonel Kearney's camp of Dragoons, to which place I had to go, although very unwell. I returned the next day, and that night received an order to go to General Leavenworth's Camp. The order was positive and must be obeyed; so the next morning I started, having first given directions to my Hospital steward how to treat the cases I left behind. When I arrived at Gen. Leavenworth's Camp, I found that officer at death's door, yet I prescribed and determined to remain all night; the next morning he was better, and I was ordered to remain; but he was too far gone, and soon commenced sinking very fast. In the night, I thought he was dying, the next morning still sinking, and in the afternoon, seeing all hope at an end, I requested leave to return, and left him about 5, P. M. He died shortly afterwards, about 50 hours from the time I first saw him. I rode late, having 25 miles to ride to the Dragoon Camp; and the next day returned to this place, bringing with me 15 sick. During my absence two deaths, one an officer, had occured; and two more were so far gone as to render hope but a dream. A few days afterwards, 27 additional sick arrived, making 125 on the report. I alone for duty. My duties then, as now, were too great. I feel myself fast sinking under them; but I am the only hope. I yesterday had nearly given over, and last night made a written application to Col. Kearney for assistance. Out of six officers here, I am the only one fit for duty, and have, therefore, much devolving upon me. I am Surgeon, Quarter Master, Commissary, and Commanding Officer; but trouble myself about none but the first. The Fever is assuming a more malignant form, than it has heretofore borne, and will indeed, must prove very fatal. Our situation is pitiable. Encamped along a small creek, without regard to order or defence, in a country were the summer sun withers the grass at noon, and the cold night breeze chills one when wrapped in a cloak; with 112 on the sick report, and but a pitiful supply of medicine, and still worse supply of cooking utensils, to prepare the diet of the sick, who are lying on prairie grass, with only two blankets, one to keep the dampness from the ground, the other in the night to cover and protect them, from the cold; who can expect to treat diseases with success, or who, when attacked, can expect to recover. I have witnessed scenes of misery and woe, but never have I witnessed any thing to equal this. I have urged repeatedly, that this Camp should be removed, but the Colonel says it cannot be done. The men, when once they recover, are, with few exceptions, re-attacked. One more effort I will make for this Camp to be removed, and if it is not, then I must submit. But it is discouraging, indeed, so much so, that I now take but little pleasure in prescribing; and, although I feel that the lives of many depend upon my bearing up, yet I cannot feel cheerful nor hardly attempt to cheer up others. I have been writing until I am fatigued, and have now, the second time to day, to examine and prescribe for 112 men, besides 5 officers, though I feel like lying down myself."

Shortly after this letter was written, Dr. Hales was taken down with the prevailing fever. His attack was protracted, and terminated in dysentery, which assumed a chronic form. With the return of the expedition he was brought back to Fort Gibson, where he lingered till the 30th of the succeeding January, when he expired; having in the brief period of his early professional life, performed a greater amount of arduous duty, and endured greater sufferings, privations and anxieties, than fall to the lot of the private practitioner in the course of several years.

